# 

**Published:** 1909-01

**Authors:** 


### BOOKS RECEIVED.

The National Standard Dispensatory—containing the National History, Chemistry, Pharmacy, Actions and Uses of Medicines, including those recognised in the Pharmacopeias of the United States, Great Britain and Germany, with many references to other foreign pharmacopeias. In accordance with the Eighth Decennial Revision of the U. S. Pharmacopeia, by authorisation of the convention. By Hobart Amory Hare, B.Sc., M.D., Professor of Therapeutics and Materia Medica in Jefferson Medical College, Philadelphia; Charles Caspari, Jr., Ph.G., Phar. D., Professor of Theoretical and Applied Pharmacy in the Maryland College of Pharmacy, Baltimore; and Henry H. Rusby, M.D., Professor of Botany and Materia Medica in the College of Pharmacy of the City of New York; Members of the Committee of Revision of the U. S. P.; New (2d) edition, thoroughly revised. Imperial octavo, 2050 pages, with 478 engravings. Cloth, $6.00, net; full leather, $7.00, net. Thumb-letter index, 50 cents extra. Lea & Febiger, Publishers, Philadelphia and New York.

Modern Medicine. Its Theory and Practice. In original contributions by American and Foreign authors. Edited by William Osler, M.D., Regius Professor of Medicine in Oxford University,

England; formerly Professor of Medicine in Johns Hopkins University, Baltimore; in the University of Pennsylvania, Philadelphia, and in McGill University, Montreal. Assisted by Thomas McCrea, M.D., Associate Professor of Medicine and Clinical Therapeutics in Johns Hopkins University, Baltimore. In seven octavo volumes of about 900 pages each, illustrated. Volume V., Diseases of the Alimentary Tract. Price per volume: cloth, $6.00, net; leather, $7.00, net; half morocco, $7.50, net. Lea & Febiger, Publishers, Philadelphia and New York. 1908.

Progressive Medicine, Vol.X., No.4, December 1908. A Quarterly Digest of Advances, Discoveries and Improvements in the Medical and Surgical Sciences. Edited by Hobart Amory Hare, M.D., Professor of Therapeutics and Materia Medica in the Jefferson Medical College of Philadelphia, assisted by H. R. M. Landis, M.D. Octavo, 333 pages, with 28 engravings. Lea & Febiger, Publishers, Philadelphia and New York. (Per annum, in four cloth-bound volumes, $9.00; in paper binding, $6.00, carriage paid to any address.)

Transactions of the American Otological Society. Forty-first annual meeting, held at Atlantic City, N. J., June 23, 24, 1908. Vol. XI., part 1. Edited by Dr. J. F. McKernon, Secretary, 62 W. 52d St., New York. Published by the Society. Mercury Publishing Co., Printers, New Bedford, Mass. 1908.

Vaccine Therapy and the Opsonic Method of Treatment. A short compendium for general practitioners, students and others. By R. W. Allen, M.D., B.S. (Lond.) late pathologist to the Royal Eye Hospital, etc. Second edition. Octavo, pp. 244. Philadelphia, Pa. Blakiston's Son & Company. 1908. (Price, $2.00, net.)

Transactions of the American Surgical Association. Vol. XXVI. Edited by Richard H. Harte, Recorder. Printed for the Association by William J. Dornan, Philadelphia. 1908.

Report of the Commissioner of Education of the United States for the year ended June 30, 1907. Vol. I. By Elmer Ellsworth Brown, Ph.D., LL.D. Washington: Government Printing Office. 1908.

Cataract Extraction. By H. Herbert, F.R.C.S., late Lieutenant Colonel I. M. S., Professor of Ophthalmic Medicine and Surgery in Grant Medical College, and in charge of the Sir Cowasjee Jehangir Ophthalmic Hospital, Bombay. Otavo, pp. 391. New York: William Wood & Company. 1908.

International Clinics. A Quarterly of Illustrated Clinical Lectures and especially prepared articles on Treatment, Medicine, Surgery, Neurology, Pediatrics, Obstetrics, Gynecology, Orthopedics, Pathology, Dermatology, Ophthalmology, Otology, Rhinology, Laryngology, Hygiene and other topics of interest to students and practitioners. Edited by W. T. Longcope, M.D., Volume IV. eighteenth series. Philadelphia and London: J. B. Lippincott Co. 1908. (Cloth, $2.00.)

